# Do pictorial health warnings on waterpipe tobacco packs matter? Recall effectiveness among Egyptian waterpipe smokers & non-smokers

**DOI:** 10.1371/journal.pone.0208590

**Published:** 2018-12-18

**Authors:** Aya Mostafa, Heba Tallah Mohammed, Rasha Saad Hussein, Wafaa Mohamed Hussein, Mahmoud Elhabiby, Wael Safwat, Sahar Labib, Aisha Aboul Fotouh

**Affiliations:** 1 Department of Community, Environmental, and Occupational Medicine, Faculty of Medicine, Ain Shams University, Cairo, Egypt; 2 School of Pharmacy, University of Waterloo, Waterloo, Ontario, Canada; 3 Department of Psychiatry Medicine, Faculty of Medicine, Ain Shams University, Cairo, Egypt; 4 Egyptian Tobacco Control Coalition, Cairo, Egypt; 5 Egypt Health Foundation, Cairo, Egypt; 6 Tobacco Control Unit, Ministry of Health, Cairo, Egypt; Wake Forest University School of Medicine, UNITED STATES

## Abstract

**Introduction:**

Despite the global rise in waterpipe tobacco smoking (WTS), the effectiveness of waterpipe tobacco health warnings remain understudied, especially in countries with high WTS rates. Egypt has been employing waterpipe tobacco labelling for a decade, however, their effectiveness is unknown. Our overall aim was to measure the effectiveness of pictorial health warnings (PHWs) on waterpipe tobacco packs (WTPs) through participant memory recall and to investigate whether they induced behavioural responses in waterpipe smokers and deterred uptake of WTS in non-smokers, examining the differentials of effectiveness among socio-demographic subgroups.

**Subjects and methods:**

We conducted two surveys including 1490 adult current waterpipe smokers, 73 former waterpipe smokers, and 451 non-smokers in Cairo and a rural village in Egypt between 2015–2017. Participants who noticed PHWs on WTPs were asked questions about salience, communication of health risks, public support, cognitive processing, and self-reported behavioural responses (current waterpipe smokers: reduce consumption, forgo a smoke, quit attempts; former waterpipe smokers: quit; non-smokers: deter WTS initiation). Univariate and multivariable statistical analyses were performed.

**Results:**

Participants’ mean age was 35 years, mostly males (90.4%), waterpipe smokers (74.0%) and rural residents (59.3%). Approximately two-thirds of participants noticed PHWs on WTPs. Salience was significantly less among females, urban residents and participants with high literacy. More than three-quarters of participants reported that WTS health risks were communicated through the warnings. At least half of participants cognitively processed the warnings: 56.3% thought of the warnings when WTPs were out of sight; non-smokers understood the warnings (83.2%) and discussed them with others (90.3%) significantly more than current (76.0% and 72.5%, respectively) and former waterpipe smokers (81.0% and 61.9%, respectively). Participants reported that PHWs on WTPs motivated 58.5% of waterpipe smokers to think about quitting; 64.5% to reduce their consumption; 42.2% to forgo a smoke; 24.5% to attempt to quit; 57.1% of former waterpipe smokers to successfully quit; and 59.3% of non-smokers to remain smoke-free.

**Conclusions:**

Findings suggest that inserting PHWs on WTPs is an effective waterpipe tobacco labelling policy. Countries with similarly high rates of WTS should consider adopting WTP PHWs within a comprehensive regulatory framework.

## Introduction

Waterpipe tobacco smoking (WTS) is a rising global epidemic; recently the practice has been spreading in the West and among youth and women, while for decades it was confined to the East and older men [1–4]. For instance, a recent systematic review of studies on trends reported an annual increase in WTS among youth in the United States by up to 1% per year [5]. The World Health Organization Framework Convention on Tobacco Control (WHO FCTC) recommends the adoption of waterpipe tobacco labelling and packaging regulations as one of the cost-effective public health tools to control this morbid habit [6]. Despite this, PHWs on WTPs are only obligatory in a few countries [7] and are not mandated in most settings in the WHO Eastern Mediterranean Region (EMR), where WTS rates are the highest and have surpassed cigarette smoking in females and adolescents [8,9]. In Egypt, a comparison between the Global Youth and Adult Tobacco Surveys revealed that adolescent girls were 11 times more likely to smoke waterpipe tobacco than adult women [10]. In 2009, 6.2% of Egyptian males aged 15 years or older smoked waterpipe tobacco; rates were higher in rural (7.5%) than urban (4.9%) areas [11] In 2014, a WHO report found that 12.2% of university students in Egypt were current waterpipe smokers [12].

Current evidence on the effectiveness of pictorial health warnings (PHWs) is mainly derived from numerous cigarette pack studies [13]. PHWs on cigarette packs are effective in communicating health risks of smoking [14–17]; they stimulate quit-related cognitive responses and behaviour in smokers [17–21]; and prevent uptake of smoking in non-smokers [13,22–25]. Yet, health warning labelling of non-cigarette tobacco products, including waterpipe tobacco, remain understudied. To our knowledge, few published studies have examined the impact of waterpipe tobacco PHWs: two online surveys from Canada and the US—both countries do not require PHWs on waterpipe tobacco packs (WTPs) [26,27], four qualitative studies from the UK, Egypt and the EMR [28, 29, 30, 31] and one survey from Egypt that explored expert and public opinion of the visibility of existing PHWs on WTPs and their best likely position on waterpipes [32]. The first two studies were experimental and tested hypothetical text-only versus PHWs, with limited effect on established waterpipe users. However, PHWs improved respondents’ knowledge about health hazards of WTS. The nature of the qualitative studies did not allow for estimates of effectiveness to be drawn, however, they suggested PHWs on WTPs or on the waterpipe devices could potentially communicate health risks, deter initiation and promote cessation of WTS. The Egyptian survey explored salience of PHWs on WTPs; behavioural outcomes in waterpipe smokers and non-smokers were not measured.

Egypt was one of the first countries in the EMR to introduce a textual health warning on all tobacco products in 1981, 24 years prior to ratifying the WHO FCTC [33]. In 2002, the textual warning was required to occupy at least 30% of the main display areas of the tobacco pack. By 2008, in line with the WHO FCTC guidelines, Egypt employed a set of four generic PHWs that appear on the bottom 50% of the front and back of all tobacco packs; these PHWs carry relevant textual warnings, the quitline number (16805) with an additional standard textual warning beneath it: “smoking damages health and causes death”; and are rotated every two years [33]. However, WTPs still depict colourful fruits and flavours using brand imagery. The sides of WTPs display information on the name and contacts of the manufacturer, barcode, weight and price of the WTPs.

After a decade of employing PHWs on WTPs, their effectiveness remains unknown. We aimed in this study to measure the effectiveness of PHWs on WTPs through participant recall of salience; communication of WTS health risks; public support of the warnings; and cognitive processing of the warning messages. We investigated whether PHWs on WTPs induced actual self-reported behavioural responses in waterpipe smokers (reduce consumption, forgo a smoke, motivate to quit, quit attempts, quit), and deterred WTS uptake in non-smokers. We also examined the possible differentials of effectiveness of PHWs on WTPs among various socio-demographic subgroups in this study.

## Subjects and methods

### Ethics statement

This study was approved by the Ethical Review Board of Faculty of Medicine, Ain Shams University (FMASU R 10/2015 and 10a/2016). The Ethical Review Board approved the verbal consent procedure. This study was qualified as involving no or minimal risk to participants. In addition, illiteracy is relatively high in the population (approximately 25%) [34]. Therefore, verbal consent was considered more practical in this large low-risk survey. The interviewers read aloud a written information sheet (for standardization of the information conveyed to all potential participants) including an introduction of the research team, the goals of the study and its expected public health benefit, the time required to complete the face-to-face interview questionnaire, and that: enrolment was voluntary, participants were free to withdraw from the study, confidentiality was ensured, data collected was anonymous, participants could not be identifiable as they were assigned serial identification numbers, and data will be published collectively. Contacts of the research team in Faculty of Medicine, Ain Shams University were provided to all potential participants in case they had any questions. Then, individuals were asked whether they agreed to participate in the study. In case of willingness to participate, consent was given verbally before starting the interview and recorded by ticking a box in the paper form of the questionnaire. Participants were not provided any incentives for completing the study. The Ethical Review Board approved this verbal consent procedure.

### Design

**Fig 1:**
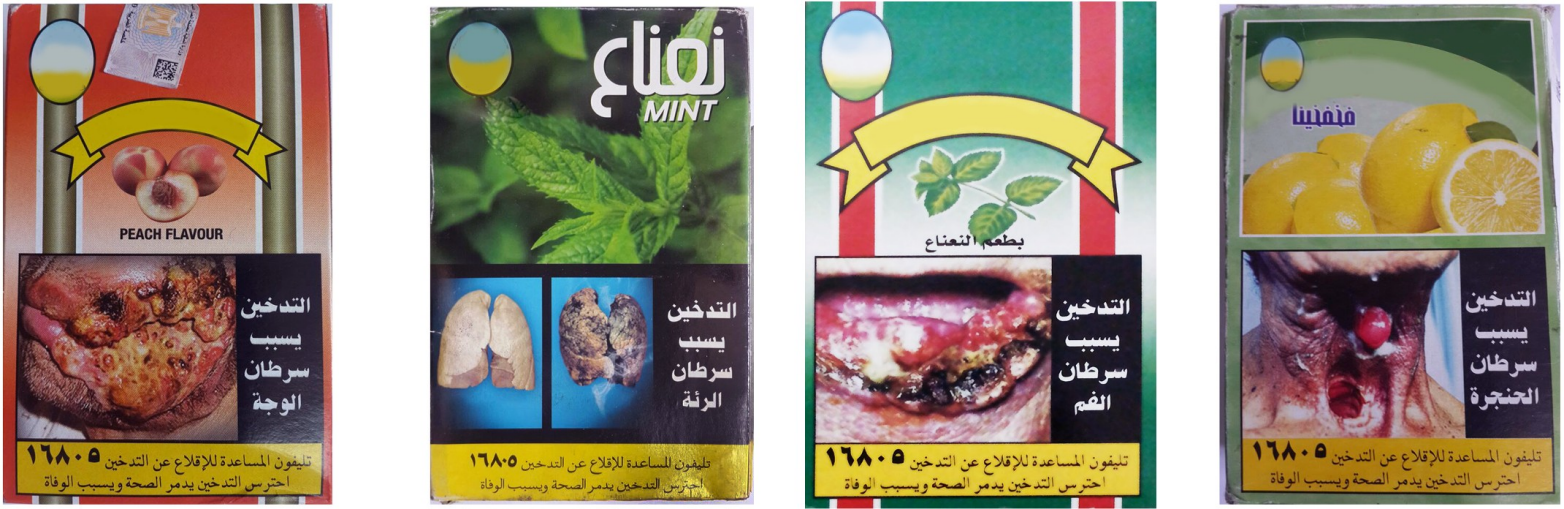
Existing PHWs in the Egyptian market at the time of survey round 1. Textual warnings associated with PHWs: Smoking causes…(A) Face cancer (B) Lung cancer (C) Mouth cancer (D) Throat cancer Reprinted under a CC BY license, with permission from the WHO, original copyright [2014].

**Fig 2:**
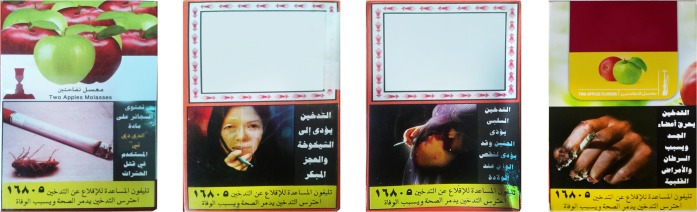
Existing PHWs in the Egyptian market at the time of survey round 2. Textual warnings associated with PHWs: (A) Cigarettes contain “DDT” that is used in killing insects (B) Smoking leads to early aging and disability (C) Passive smoking harms the fetus and may lead to reduced weight at birth (D) Smoking burns body organs and causes cancers and cardiac diseases. Reprinted under a CC BY license, with permission from the WHO, original copyright [2016].

This study consisted of two rounds of surveys, where each assessed the inherent effectiveness of the existing PHWs on WTPs through participants’ memory recall. Standard study procedures were applied by the same interviewers in both survey rounds as regards the sampling approach, sample size, survey tool, and administration methods. The study spanned two PHW rotations, so we could assess two different sets of PHWs **(Figs 1 and 2)**. Survey round 1 (June through November 2015) was conducted 14–19 months after the introduction of the first PHW set in April 2014. Survey round 2 (September 2016 through January 2017) was conducted 5–10 months after the introduction of the second PHW set in April 2016.

### Sample

For each survey round, we targeted a sample of 1025 waterpipe smokers and non-smokers aged 18 years or older in urban Cairo, and a rural village in Menoufia governorate. The sample size was based on a one-sided 95% lower-limit confidence interval with a distance from the sample proportion to the lower limit = 0.010 when the sample proportion = 0.040 (assuming a 4% change in smoking behaviour in the general population), alpha level = 0.05, and power = 0.8.The calculated sample size was 932 individuals for each survey round, then we added 10% (n = 93) to accommodate for possible missing data.

We used purposive quota non-random sampling to recruit individuals representing national proportions of: gender (51% males and 49% females); age-group (30% of 18–24 years old and 70% of 25+ years old); residence (40% urban and 60% rural); and WTS status (in those above 15 years old: 93.8% non-smokers and 6.2% waterpipe smokers). Since we were not aiming to estimate the prevalence of WTS in this study and our primary interest was evaluating the effectiveness outcomes of WTP PHWs (which were more in waterpipe smokers (e.g. motivate to quit, reduce consumption, forgo a smoke) versus one outcome in non-smokers (deterring WTS uptake)), we oversampled waterpipe smokers recruiting approximately 75% waterpipe smokers and 25% nonsmokers. Accordingly, we handed the field supervisor a table with the requested numbers per the aforementioned demographic categories and WTS status. We closely monitored the proportions enrolled according to this sampling scheme during the 5-month data collection period for each survey round. The field interviewer handed in a monthly report with actual numbers enrolled per each category. The final sampling proportions were: gender (90.4% males and 9.6% females); age-group (36.9% of 18–24 years old and 63.1% of 25+ years old); residence (40.7% urban and 59.3% rural); and WTS status (74.0% waterpipe smokers and 26.0% non-smokers).

Trained field interviewers approached different individuals in each survey round at cafes, households, workplaces, and universities and explained the purpose of the study before they screened individuals for age eligibility; the only eligibility criterion was being 18 years of age or older. We had a list of all districts of Cairo, which are officially categorized almost equally into urban and semi-urban neighborhoods. Households in these neighborhoods and in the rural village in Menoufia were approached in a ‘door-to-door’ fashion where the interviewer knocked on random houses. We included a variety of different population sources to decrease potential bias in recruiting participants from a single source population. Individuals were not known to the interviewers before the study to avoid potential selection bias. Individuals who agreed to participate in the study provided their verbal consent and were assigned a serial identification number for anonymity, then completed a 25-minute face-to-face interview questionnaire. Participants in survey round 1 were different from participants in survey round 2. However, there were no significant differences between participants’ demographic characteristics across the two survey rounds. The final total sample recruited in both survey rounds included 2014 participants.

### Measures

Survey questions were developed based on published literature on effectiveness of PHWs [35–37] and adapted to be waterpipe-specific. The questionnaire was originally developed in English, then translated into Egyptian colloquial Arabic that was back-translated to English for validation and was pilot tested. The measures included in the questionnaire were tested for face and content validity by two tobacco control experts. In addition, we pilot tested these measures for appropriateness in the waterpipe context. Pilot testing involved 57 participants (24 in urban Cairo 33 in rural Menoufia), who were 19–76 years old (mean age 39.2±14.4); 47 (82.5%) were males and 10 (17.5%) were females; 42 (73.7%) were waterpipe smokers and 14 (26.3%) were non-smokers. Pilot data were not included in this analysis. During the pilot phase, we prepared a manual of operations and detailed instructions for field work and tested the interviewers’ adherence to these. The questionnaire was printed in paper forms. The interviewers read the survey questionnaire to participants and were trained to adhere to the wording, sequence of order and skipping instructions of the questionnaire. We chose this approach in administering the questionnaire because the illiteracy rate in the population is high (approximately 25%) [34] and to maximize completeness of responses. Comments from participants, interviewers, data entry personnel, and the research team were taken into consideration while developing the final version of the questionnaire that was used in both survey rounds.

The questionnaire included several sections; we focus in this article on presenting the part related to recall effectiveness of PHWs on WTPs. Respondents were asked about their WTS history. They were then divided into those who had never smoked (never smokers) and those who had smoked waterpipe (ever waterpipe smokers), who were further split into current or former waterpipe smokers (depending on whether they had smoked waterpipe at least once within the past month) [38]. Socio-demographic data included age, gender, education, occupation, marital status, and urban/rural residence. Measures of PHW recall effectiveness included: salience, cognitive processing, communication of health risks. The behavioural responses were to remain smoke-free (non-smokers); decrease consumption (i.e. smoking fewer sessions or fewer hagars/portion of waterpipe tobacco per session), forgo a smoke, induce quit attempts (current waterpipe smokers), and quit waterpipe (former waterpipe smokers); avoidance and public support of PHWs (all participants).

*Salience* of warning labels on WTPs was measured by asking participants: “Are you aware of health warnings on WTPs?” (yes/no). If so, participants were asked: “Did you notice that warnings contained pictorials?” (yes/no/don’t know), and, “How often did you read or look closely at the warnings?” (4-point scale: not at all, a little, somewhat, a lot). Other aspects of salience were assessed to ensure actual warning exposure: whether participants reported that the PHWs on WTPs were different from those on cigarette packs, whether participants noticed that the PHWs included the hotline for smoking cessation, whether participants could correctly recall the position of PHWs on the WTP and whether participants knew the information on the side of WTPs.

We measured *communication of health risks* by asking participants who noticed PHWs, if at all: “Did the warnings on WTPs increase your knowledge about health risks of WTS?”; “Overall, how good or bad is WTS for your health? (good, neither good or bad, bad, don’t know); “When compared to cigarette smoking, WTS is: less harmful, more harmful, about the same, don’t know?”; and “When compared to cigarettes, waterpipe contains: less nicotine, more nicotine, about the same, don’t know?”

*Cognitive processing* was assessed by asking those who noticed PHWs: “Were the warnings understandable?” (yes/no); “Have you ever thought about the warnings even when the WTP was not in sight?”, and “Have you talked about or discussed the warnings on WTPs with others?” (4-point scale: never, rarely, sometimes, many times). Also, if at all, “Did the warning labels on WTPs make you think/worry about the health risks of WTS?”.

*Public support* was measured by asking respondents: “Do you agree with having warning labels on WTPs?” (yes/no); “Do you agree that they contain information on cessation of WTS?” (yes/no); “Do you think the information that the warnings communicate about health risks (compared to current information) should be: less, same, more?”; and “Do you think the warnings’ size (compared to current size) on the WTP should be: smaller, same, larger?”

For recording *self-reported behavioural outcomes*, we asked current waterpipe smokers who noticed PHWs, if the warning labels on WTPs: “made you reduce the number of hagars you smoke?”; “led you to decide not to have a waterpipe smoke when you were about to?” (never, rarely, sometimes, many times); “Did the warning labels on WTPs motivate you to quit?” (not at all, somewhat, a lot). We also asked current waterpipe smokers who noticed PHWs: “How many times were you able to quit WTS for more than one week?” (never, once, many times)”; “Do you intend to quit WTS?” (not at all/ yes: within the current month, next 6 months, in the future); and self-efficacy “Do you believe you could quit WTS at any time you want to?” (yes/no). Former waterpipe smokers who noticed PHWs were asked: “What were the reason(s) you quit WTS?” (more than one answer option was allowed in this combined closed- and open-ended question a) yes/no for each of the following: health problems, cost, warnings on cigarette packs, warnings on WTPs, advertisements on television or through other media, for the sake of family, and b) other reasons), and how long since they had quit (a month, 2–5 months, 6–11 months, a year or more). We asked non-smokers who noticed PHWs: “Did warnings on WTPs prevent you from starting to smoke waterpipe?” (yes/no). We asked both waterpipe smokers and non-smokers who noticed PHWs: “Did you avoid the warnings on WTPs?” (yes/no).

### Analysis

Data were analysed using the Statistical Package for Social Sciences (SPSS) (SPSS; IBM Corp, Armonk, NY. Version 22; 2013). Descriptive analysis of quantitative data was performed to obtain means, standard deviations and frequencies. The 4-point scale responses were dichotomized; “never” and “rarely” or “not at all” and “a little” into “no” versus “sometimes” and “many times” or “somewhat” and “a lot” into “yes”. “Don’t know” and missing responses were reported where available. Results are based on the combined sample for both survey rounds (n = 2014). In this analysis, “smokers” refers to current waterpipe smokers (n = 1490), “non-smokers” refers to never-users of waterpipe tobacco (n = 451). Former waterpipe smokers (n = 73) refers to participants who reported they quit WTS and indicated they did not smoke waterpipe in the past month. Bivariate analysis of the sample who reported noticing PHWs on WTPs (n = 1261) was performed using the chi-square test for categorical variables. Logistic regression analyses of the same sample (n = 1261) were conducted to identify factors associated with change in current waterpipe smokers’ behaviour (reduce consumption; forgo a smoke; quit attempt); having successfully quit in former waterpipe smokers; and remaining smoke-free in non-smokers. Salience; communication of health risks; cognitive processing; and public support were introduced into the model as composite variables, where a positive response to any question listed here above under each of these measures was used as an indicator of its corresponding overarching measure. Models were adjusted for gender; age; educational attainment; occupation; marital status; urban/rural residence; and frequency of WTS. Adjusted odds ratios (AOR) and 95% confidence intervals (CI) were reported. A p-value of <0.05 was considered statistically significant.

## Results

### Sample characteristics

Our total sample included 2014 participants (response rate 98.2%); 1015 and 999 completed survey rounds 1 and 2, respectively. 679 (33.7%) were met at cafes, 436 (21.6%) at households, 498 (24.7%) at workplaces, and 401 (20.0%) at universities. Participants’ mean age was 35 years and they were mostly males (90.4%), current waterpipe smokers (74.0%), married (63.1%), rural residents (59.3%), attained at least high school education (80.5%), and skilled workers (61.1%). Females, professionals or students/unemployed, participants with higher educational attainment, and urban residents were significantly less likely to notice PHWs on WTPs compared to their counterparts **Table 1**.

**Table 1:** Socio-Demographics and waterpipe tobacco smoking status of participants by noticing PHWs on WTPs, survey rounds 1 and 2, in 2015–2017, Egypt.

	**Total**	**Noticed PHW**		p-value*
		**Did**	**Did not**	
	**N = 2014**	**N = 1261**	**N = 753**	
	**(column%)**	**(row%)**	**(row%)**	
**Gender**				
Male	1820 (90.4)	1230 (67.6)	590 (32.4)	
Female	194 (9.6)	31 (16.0)	163 (84.0)	**<0.001**
**Age,** Mean±SD	35.2±13.6	35.3± 13.7	34.9 ±13.3	0.646**
**Age group**				
18–24	743 (36.9)	479 (64.5)	264 (35.5)	
25+	1271 (63.1)	782 (61.5)	489 (38.5)	0.198
**Education**				
Low	392 (19.5)	291 (74.2)	101 (25.8)	
Moderate	679 (33.7)	576 (84.8)	103 (15.2)	
High	943 (46.8)	394 (41.8)	549 (58.2)	**<0.001**
**Occupation**				
Professional	314 (15.6)	122 (38.9)	192 (61.1)	
Skilled	1230 (61.1)	894 (72.7)	336 (27.3)	
Student, unemployed	470 (23.3)	245 (52.1)	225 (47.9)	**<0.001**
**Marital status**				
Unmarried	743 (36.9)	431 (58.0)	312 (42.0)	
Married	1271 (63.1)	830 (65.3)	441 (34.7)	**0.001**
**Residence**				
Urban	820 (40.7)	105 (12.8)	715 (87.2)	
Rural	1194 (59.3)	1156 (96.8)	38 (3.2)	**<0.001**
**Waterpipe tobacco smoking**				
Non-smoker	451 (22.4)	268 (59.4)	183 (40.6)	
Former smoker	73 (3.6)	42 (57.5)	31 (42.5)	
Current smoker	1490 (74.0)	951 (63.8)	539 (36.2)	0.157
Daily	1210 (81.2)	816 (67.4)	394 (32.6)	
Non-daily	280 (18.8)	135 (48.2)	145 (51.8)	**<0.001**

* Chi-squared test, p-values < 0.05 indicate differences in noticing PHWs within groups

** Independent samples T-test

### Salience of PHWs on WTPs

Almost two-thirds of current waterpipe smokers (63.8%), former waterpipe smokers (57.5%) and non-smokers (59.2%) noticed pictorials in the health warning labels on WTPs, among these most (81.6%) read or looked closely at the PHWs. Most participants (88.7%) knew that PHWs on cigarette packs were identical to those on WTPs and noticed the hotline number for cessation (88.7%). Almost all participants recalled the position of the PHW correctly (97.7%), but only 1.1% knew the information written on the side of the packs **Table 2**.

**Table 2:** Salience, communication of health risks, cognitive processing, and public support of PHWs on WTPs, survey rounds 1 and 2, in 2015–2017, Egypt.

	**Total**	**Current Smoker**	**Former Smoker**	**Non-smoker**	
**Salience**	**N = 2014 (%)**	**N = 1490 (%)**	**N = 73 (%)**	**N = 451 (%)**	p-value*
***Aware of health warnings on packs***					
*Yes*	1298 (64.4)	974 (65.4)	47 (64.4)	277 (61.4)	0.308
***Noticed pictorials***					
*Yes*	1261 (62.6)	951 (63.8)	42 (57.5)	268 (59.4)	0.144
***Read/ looked at PHW closely***	**N = 1261**	**N = 951**	**N = 42**	**N = 268**	
*Sometimes*, *A lot*	1029 (81.6)	770 (81.0)	31 (73.8)	228 (85.1)	0.128
***Same as cigarette pack warnings***					
*Yes*	1118 (88.7)	841 (88.4)	36 (85.7)	241 (89.9)	0.700
*Missing*	8 (<1.0)	5 (<1.0)	1 (2.4)	2 (<1.0)	
***Noticed hot line number***					
*Yes*	1118 (88.7)	854 (89.8)	32 (76.2)	232 (86.6)	**0.018**
*Missing*	5 (<1.0)	4 (<1.0)	1 (2.4)	0	
***PHW position recalled correctly***					
*Yes*	1232 (97.7)	926 (97.4)	40 (95.2)	266 (99.3)	0.107
***Know information on pack sides***					
*Yes*	14 (1.1)	12 (1.3)	1 (2.4)	1 (0.4)	0.341
*Missing*	4(<1.0)	4 (<1.0)	0	0	
**Communication of health risks**	**N = 1261 (%)**	**N = 951 (%)**	**N = 42 (%)**	**N = 268 (%)**	p-value*
***Overall perception of WTS***					
*Bad*	1131 (89.7)	836 (87.9)	31 (73.8)	264 (98.5)	**<0.001**
*Don’t know*	12 (1.0)	8 (<1.0)	3 (7.1)	1 (<1.0)	
***Increased knowledge of health hazards***					
*Somewhat*, *A lot*	956 (75.8)	707 (74.3)	36 (85.7)	213 (79.5)	0.070
***Waterpipe tobacco compared to cigarettes***					
*More harmful*	1015 (80.5)	743 (78.1)	28 (66.7)	244 (91.0)	**<0.001**
*Don’t know*	7 (<1.0)	6 (<1.0)	0	1 (<1.0)	
***Nicotine content compared to cigarettes***					
*More nicotine*	107 (8.5)	89 (9.4)	8 (19.0)	10 (3.7)	**0.003**
*Don’t know*	17 (1.3)	14 (1.5)	1 (2.4)	2 (<1.0)	
**Cognitive processing**	**N = 1261 (%)**	**N = 951 (%)**	**N = 42 (%)**	**N = 268 (%)**	p-value*
***Understandable***					
*Yes*	980 (77.7)	723 (76.0)	34 (81.0)	223 (83.2)	**0.039**
***Thought of warning when pack away***					
*Sometimes*, *Often*	710 (56.3)	534 (56.2)	26 (61.9)	150 (56.0)	0.757
***Discussed health warnings with others***					
*Sometimes*, *Often*	957 (75.9)	689 (72.5)	26 (61.9)	242 (90.3)	**<0.001**
***Made you worry about health hazards***					
*Somewhat*, *A lot*	1053 (83.5)	782 (82.2)	37 (88.1)	234 (87.3)	0.101
***Avoid looking at PHWs on WTPs***					
*Yes*	895 (71.0)	709 (74.6)	18 (42.9)	168 (62.7)	**<0.001**
*Missing*	5 (<1.0)	3 (<1.0)	0	2(<1.0)	
**Public support**	**N = 1261 (%)**	**N = 951 (%)**	**N = 42 (%)**	**N = 268 (%)**	p-value*
***Agree to have PHWs on WTPs***					
*Yes*	1176 (93.3)	868 (91.3)	41 (97.6)	267 (99.6)	**<0.001**
***Agree to include information on cessation***					
*Yes*	1136 (90.1)	839 (88.2)	37 (88.1)	260 (97.0)	**<0.001**
*Missing*	3 (<1.0)	3 (<1.0)	0	0	
***Think health risk information should be***					
*More (compared to current)*	379 (30.1)	213 (22.4)	29 (69.0)	137 (51.1)	**<0.001**
***Thinks PHW size on WTP should be***					
*Larger (compared to current)*	344 (27.3)	185 (19.5)	22 (52.4)	137 (51.1)	**<0.001**

* Chi-squared test, p-values < 0.05 indicate differences between current waterpipe smoker, former waterpipe smoker and non-smoker groups; no other covariates were included

### Communication of health risks through PHWs on WTPs

Significantly more non-smokers than current and former waterpipe smokers reported that WTS is bad for health and is more harmful than cigarette smoking. More former waterpipe smokers reported that PHWs on WTPs increased knowledge about WTS hazards than current waterpipe smokers and non-smokers, however, the difference was not statistically significant. Most participants reported that the nicotine content of waterpipe tobacco is not higher than that of cigarettes **Table 2**.

### Cognitive processing of PHWs on WTPs

Most participants reported high rates of cognitive processing of PHWs on WTPs. Non-smokers understood the warnings and discussed them with others significantly more than current and former waterpipe smokers. More than half of participants thought about PHWs even when the pack was out of sight, waterpipe smokers and non-smokers alike. Current waterpipe smokers avoided looking at PHWs on WTPs significantly more than former waterpipe smokers and non-smokers **Table 2**.

### Public support of PHWs on WTPs

Almost all (93.3%) participants supported having PHWs on WTPs and including more cessation information (90.1%), yet these percentages were significantly higher among non-smokers than current waterpipe smokers. Moreover, former waterpipe smokers and non-smokers were more likely to support inclusion of more health risk information and larger PHWs on WTPs than waterpipe smokers **Table 2**.

### Quit thoughts, intentions and other self-reported behavioural responses to PHWs on WTPs

**Fig 3:**
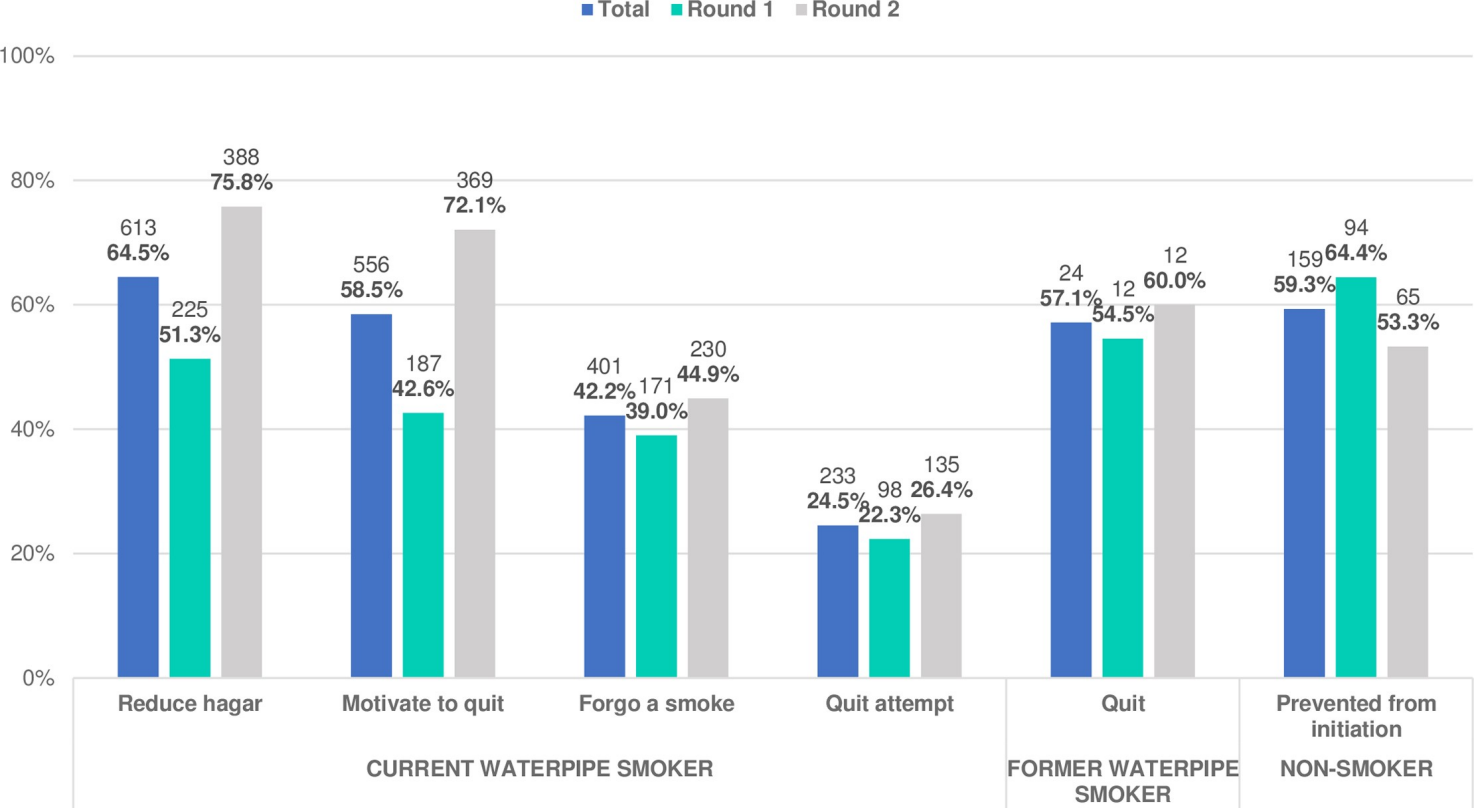
Behavioural responses among non-smokers, current and former waterpipe smokers who noticed PHWs on WTPs, survey rounds 1 and 2, in 2015–2017, Egypt.

Among current waterpipe smokers who noticed PHWs on WTPs, more than two-thirds (64.5%) reported that they reduced the number of hagars smoked due to viewing the warnings. Less than half (42.2%) reported that they forgone a smoke sometimes or many times due to viewing the PHW on WTPs, and only a quarter (24.5%) reported that the warnings encouraged them to make at least one quit attempt **(Fig 3)**. Of the current waterpipe smokers, 58.5% reported that PHWs on WTPs motivated them to think about quitting WTS, while almost half of them (46.0%) believed they could quit WTS at any time they decided to. Most current waterpipe smokers reported they intended to quit (96.5%), but most of these (69.8%) did not decide a date for quitting.

More than half of former waterpipe smokers (57.1%) reported that PHWs on WTPs had motivated them to quit; 87.5% of these have been smoke-free for 6 months or longer. Almost two-thirds (59.3%) of non-smokers reported that PHWs on WTPs deterred them from uptake of WTS. All self-reported behavioural responses were higher in survey round 2 for current and former waterpipe smokers by 4.0–29.5% but were lower for non-smokers by 11.1% **(Fig 3)**. The highest differences between survey rounds were reported for motivation to quit (72.1% and 42.6% in surveys round 2 and 1, respectively) and reduction in consumption (75.8% and 51.3% in surveys round 2 and 1, respectively), followed by prevention of initiation (53.3% and 64.4% in surveys round 2 and 1, respectively), while smaller differences were observed for forgoing a smoke (44.9% and 39.0% in surveys round 2 and 1, respectively), quit attempts (26.4% and 22.3% in surveys round 2 and 1, respectively) and quit WTS (60.0% and 54.5% in surveys round 2 and 1, respectively) **(Fig 3)**.

### Factors associated with behavioural responses to PHWs on WTPs among non-smokers, current and former waterpipe smokers

Thinking of quitting due to PHWs on WTPs (AOR = 26.2; 95% CI:17.2–40); cognitive processing (AOR = 7.4; 95% CI:1.6–34.7); and public support (AOR = 13.4; 95% CI:3.8–47.0) were significantly and independently associated with reducing hagar consumption in current waterpipe smokers. Forgoing a smoke was independently associated with quit thoughts due to PHWs on WTPs (AOR = 11.0; 95% CI:7.5–15.7); and rural residence (AOR = 3.3; 95% CI:1.6–6.8). Quit attempts in current waterpipe smokers were independently associated with quit thoughts due to PHWs on WTPs (AOR = 2.2; 95% CI:1.4–3.4); non-daily WTS (AOR = 13.6; 95% CI:8.1–22.9); urban residence (AOR = 4.3; 95% CI:2.0–8.9); and being unmarried (AOR = 2.0; 95% CI:1.1–3.8) **Table 3**.

**Table 3:** Factors associated with self-reported behavioural responses relevant to PHWs on WTPs reported by non-smokers, current and former waterpipe smokers, survey rounds 1 and 2, in 2015–2017, Egypt.

	Current waterpipe smokers			Former waterpipe smokers	Non-smokers
	Reduce hagar	Forgo a smoke	Quit attempt	Quit	Deter uptake
N of those who noticed PHWs on WTPs	N = 613	N = 401	N = 233	N = 42	N = 268
AOR (95% CI)*					
**Gender**					
Male	1.0	1.0	1.0	1.0	1.0
Female	2.0 (0.7–5.9)	1.5 (0.5–4.9)	3.1 (0.7–13.1)	1.0 (0.4–2.2)	0.9 (0.3–2.3)
*p-value*	0.217	0.507	0.119	0.909	0.782
**Age group**					
18–24	1.0	1.0	1.0	1.0	1.0
25+	1.0 (0.5–1.8)	0.8 (0.9–1.5)	1.1 (0.6–2.0)	1.7 (0.8–3.7)	1.1 (0.7–1.6)
*p-value*	0.938	0.760	0.805	0.188	0.710
**Educational attainment**					
Low	1.0	1.0	1.0	1.0	1.0
High	2.0 (1.0–4.0)	1.6 (0.9–2.7)	2.9 (1.6–5.4)	1.8 (0.9–3.5)	0.8 (0.5–1.1)
*p-value*	0.061	0.077	**<0.001**	0.087	0.185
**Occupation**					
Other	1.0	1.0	1.0	1.0	1.0
Skilled	1.5 (0.8–3.0)	0.9 (0.5–1.6)	1.6 (0.9–3.1)	1.3 (0.7–2.4)	1.1 (0.7–1.7)
*p-value*	0.246	0.768	0.131	0.418	0.555
**Marital status**					
Unmarried	1.0	1.0	1.0	1.0	1.0
Married	1.3 (0.7–2.5)	1.0 (0.6–1.6)	0.5 (0.3–0.9)	1.0 (0.5–2.2)	0.6 (0.4–1.0)
*p-value*	0.367	0.912	**0.027**	0.914	**0.037**
**Residence**					
Urban	1.0	1.0	1.0	1.0	1.0
Rural	2.2 (0.9–5.0)	3.3 (1.6–6.8)	0.2 (0.1–0.5)	0.5 (0.2–1.1)	8.3 (4.7–14.7)
*p-value*	0.072	**0.001**	**<0.001**	0.073	**<0.001**
**Frequency of WTS**				-	-
Nondaily	1.0	1.0	1.0		
Daily	1.7 (0.9–3.2)	1.5 (0.9–2.3)	0.1 (0–0.1)		
*p-value*	0.128	0.112	**<0.001**		
**Salience**					
No	1.0	1.0	1.0	1.0	1.0
Yes	5.6 (0.3–110.4)	1.3 (0.1–15.7)	9.2 (0.5–152.6)	0.7 (0.1–6.4)	0.2 (0–1.3)
*p-value*	0.259	0.838	0.123	0.729	0.088
**Communication of health risks**		-			
No	1.0		1.0	1.0	1.0
Yes	3.7 (0.6–24.5)		0.3 (0.1–0.8)	0.6 (0.1–3.8)	2.0 (0.6–6.8)
*p-value*	0.171		**0.019**	0.623	0.246
**Cognitive processing**		-			
No	1.0		1.0	1.0	1.0
Yes	7.4 (1.6–34.7)		1.1 (0.4–2.9)	1.0 (0.2–5.2)	50.0(11.5–217.1)
*p-value*	**0.012**		0.917	0.994	**<0.001**
**Motivate to quit (Quit thoughts)**				-	-
No	1.0	1.0	1.0		
Yes	26.2 (17.2–40)	11.0 (7.7–15.7)	2.2 (1.4–3.4)		
*p-value*	**<0.001**	**<0.001**	**0.001**		
**Public support**		-			
No	1.0		1.0	1.0	1.0
Yes	13.4 (3.8–47.0)		0.6 (0.2–1.4)	4.5 (0.5–43.5)	7.5 (3.4–16.4)
*p-value*	**<0.001**		0.205	0.190	**<0.001**

*Models were adjusted for gender; age; educational attainment; occupation; marital status; urban/rural residence; and frequency of WTS

In former waterpipe smokers, none of the tested variables in the adjusted logistic regression model were significantly associated with abstinence, while in bivariate analysis, being 25 years of age or older (OR = 1.9; 95% CI:1.1–3.2; p-value = 0.032); high educational attainment (OR = 1.8; 95% CI:1.1–2.9; p-value = 0.020); and urban residence (OR = 1.8; 95% CI:1.1–2.8; p-value = 0.020) were positively associated with having successfully quit WTS due to PHWs on WTPs. In non-smokers, independent factors associated with deterring WTS uptake due to PHWs on WTPs were cognitive processing (AOR = 50.0; 95% CI:11.5–217.1); public support (AOR = 7.5; 95% CI:3.4–16.4); being a rural resident (AOR = 8.3; 95% CI:4.7–14.7); and unmarried (AOR = 1.6; 95% CI:1.0–2.6) **Table 3**.

## Discussion

We unravel in this study for the first time the effectiveness of PHWs on WTPs and examine self-reported established behavioural responses in current and former waterpipe smokers, as well as non-smokers in a country where waterpipe tobacco labelling has been employed for a decade. Non-smokers were significantly more likely to cognitively process, support and be receptive to WTS risk through PHWs on WTPs than were waterpipe smokers. Participants in our study reported that PHWs on WTPs motivated positive change in the WTS behaviour of at least half of the current waterpipe smokers and helped more than half of former waterpipe smokers to quit and non-smokers to remain smoke-free.

We also identified significant differences in effectiveness measures between various socio-demographic subgroups. About two-thirds of participants were aware of PHWs on WTPs, whereas noticeability of cigarette warning labels ranged widely in different low- to high-income countries from as low as 5% up to 80% [39]. In our study, salience was significantly lower among females, professionals or students/unemployed and participants with high educational attainment. These subgroups were mainly non-smokers, of whom only a quarter noticed PHWs on WTPs. Previous studies have similarly found that PHWs had stronger impact on smokers of lower literacy [40–43]. Notably, the majority (87.2%) of urban participants did not notice PHWs on WTPs, indicating the different practices of WTS between rural and urban regions of Egypt. Rural residents are accustomed to smoking waterpipe mainly at their homes, hence are exposed to PHWs on WTPs during preparation of waterpipe tobacco. In contrast, city residents who prefer smoking waterpipes at cafes, where waterpipe tobacco is usually prepared by waiters away from customers and is ready served, are less likely to be exposed to PHWs because WTPs may not be seen.

In our study, non-smokers were more likely to report higher knowledge about WTS hazards through PHWs on WTPs; that WTS is bad for health; and that WTS is more harmful than cigarette smoking than waterpipe smokers. Warning labels are an effective tool for communication of smoking health risks [16,44], which may in turn result in cessation behaviour in smokers and the choice of not smoking in non-smokers [15,17,20,22,23]. A meta-analysis of experimental studies reported that non-smokers usually rated warnings as being more effective than did smokers [19]. Non-smokers seem to process warnings with heightened perceived risk that reinforce continuing to be smoke-free and with more cognitive believability than smokers [18]. However, we report an important gap in risk perception related to the minimal awareness of the nicotine content of waterpipe tobacco compared to cigarettes among our study participants (8.5%), although this was not a target of any of the warnings studied in this research; similar findings were reported by Islam and colleagues [27], which reflects the need for more informative tobacco constituent labelling [45].

In agreement with the existing evidence from cigarette warning effectiveness studies [18,20,46], most of our participants reported that PHWs on WTPs elicited high rates of cognitive processing, especially in non-smokers. Cognitive reactions to warning labels are key mediators of subsequent behavioural responses [18,19,47]. We found a highly significant association between discussing warning labels with others and being smoke-free, as reported by Thrasher et al., 2016, indicating a need to enhance this element during the design of PHWs and to identify ways to utilize it for improved outcomes [14]. Also, consistent with these studies, we report that quit-related cognitive reactions were strongly and independently associated with change in the WTS behaviour (reduction of consumption, forgoing a smoke, quit attempts) of waterpipe smokers. In addition, participants reported that PHWs on WTPs motivated about one-fifth of waterpipe smokers to think about quitting and made one-third worry about health risks of WTS “a lot”, where similar rates were reported by only five middle-income countries who employ PHWs on cigarette packs [39]. Moreover, avoiding warning labels was positively associated with waterpipe smokers’ behavioural change in bivariate analyses, suggesting that this defensive reactance mediates the intended effects from viewing PHWs [20,47–49].

Public support is an important indicator of warning label effectiveness [35]. Our study reports very high levels of support (93.3%) for having PHWs on WTPs among both waterpipe smokers and non-smokers in agreement with previous cigarette on-pack pictorial warning studies [49–52]. Most participants recommended including more cessation information, in line with previous research that suggested combinations of PHWs and quit know-how text messages on cigarette packs to potentiate positive behavioural outcomes [53], inclusion of more health risk information [39], and larger PHWs on WTPs [50,54,55].

More than two-thirds of waterpipe smokers reported they have reduced their consumption of hagars due to viewing PHWs on WTPs; about two-fifths reported they have forgone a smoke due to viewing the PHWs on WTPs; and a quarter reported the PHWs on WTPs helped them try to quit. Similar high rates of positive change in smoking behaviour were noted in Canada and Australia, where PHWs have motivated about a third to two-fifths of cigarette smokers to quit [56] or make a quit attempt [21]. Forgoing a cigarette smoke is a strong indicator of subsequent quit behaviour [20,46]. However, we suggest that in the case of WTS, reduction of consumption is of equivalent or even superior importance to forgoing a smoke. If waterpipe smokers reduce their consumption by either smoking fewer sessions or fewer hagars per session, this will lead to a significant reduction in the amount of nicotine and toxicants inhaled, which have been reported to be much higher during a waterpipe smoking session compared to a cigarette smoke [45]. Moreover, more than half of former waterpipe smokers reported that PHWs on WTPs helped them to quit and most of whom have been smoke-free for 6 months or longer. Previous surveys have also found that PHWs helped about two thirds of former cigarette smokers maintain long-term abstinence [21]. Previous studies have assessed the change in cigarette smoking behaviour over time using longitudinal designs [20,45,56], while we conducted cross-sectional surveys to assess the change in current WTS behaviour as an indicator of recall effectiveness of PHWs.

Respondents reported that PHWs on WTPs were helpful also in deterring a significant proportion (59.3%) of non-smokers from uptake of WTS. These findings are consistent with non-smoker reports of warnings preventing them from taking up cigarette smoking in Canada (one-fifth) [26], the EU (one-third) [57], Australia (two-thirds) [21], and the UK (90%) [24]. We note that adults are still susceptible to waterpipe tobacco use [8], as the mean age of initiating WTS among participants of our study was 18.3±3.5 (18.0±3.2 for males and 21.9±4.5 for females) and ranged from 11 to 40 years old. Interestingly, being a rural resident was independently associated with deterring WTS uptake in non-smokers and forgoing a smoke in waterpipe smokers, while living in urban areas was associated with trying to quit. The majority of non-smokers in our study lived in rural areas, where salience was 8 times that in urban areas, which may partly explain how rural residents were more likely react to PHWs; in this case, not to smoke. Urban waterpipe smoker participants were mostly higher educated than their rural counterparts; salience, communication of WTS risks, cognitive processing, and quit thoughts were about 4-fold higher in this subgroup, which may explain the potential link with attempting to quit. Also, non-daily WTS was positively associated with quit attempts; non-daily waterpipe smokers were mostly higher educated individuals. High educational attainment was also significantly and independently associated with quit attempts in waterpipe smokers; this association was evident only in bivariate analysis for abstinence in former waterpipe smokers but failed to reach statistical significance in the multivariable regression model. Swayampakala and colleagues suggest that the initial impact of warning labels is stronger among smokers of lower literacy, but, over time, smokers with high education reported increased cognitive responses to the warnings [40].

Behavioural responses were higher in survey round 2 that was conducted closer to the introduction of PHWs than in survey round 1. This may be the reason for the observed higher behavioural responses in survey round 2 for current and former waterpipe smokers when warnings had their highest initial impact. There were no statistically significant differences in the demographic characteristics of participants across the two survey rounds, thus it is unlikely that this observation is due to differences in sampling. However, except for being more likely to quit and reduction of consumption, most differences were not significant; as the rotation frequency of PHWs is only 2 years, this may diminish wear-out of the effects of warnings. These findings are consistent with results from previous research, where changes in smoking-related behaviours were more pronounced in the short term and waned over time [54,58]. The opposite was observed for non-smokers, where warnings in survey round 1 were significantly more effective, which perhaps is related to the more fear-arousing topical content of PHWs that displayed a group of cancers caused by smoking in survey round 1. Non-smokers seem to process warnings with heightened perceived risk that reinforce continuing to be smoke-free [18], though recent research suggests that smokers may also strongly react emotionally and behaviourally to these threatening warnings [59].

The content of the existing warnings studied in this research were about smoking in general and not WTS in particular, although they appeared on WTPs. A recent report of the WHO recommended waterpipe-specific product labelling to improve the intended effects of the health warnings [5], as well as more recent quantitative [26,27,32] and qualitative [28,29,30, 31] research on PHWs on WTPs. Warnings with waterpipe-specific text and images may render PHWs on WTPs more believable, especially among participants who do not react affectively to PHWs or are resistant to changing their WTS habits or less concerned about WTS health hazards. The waterpipe-specific imagery content is important in communicating the warning message to low-literacy individuals, especially in a country like Egypt, where illiteracy rates reach up to 25% [34], while individuals with higher education may perceive PHWs on WTPs as more persuasive if accompanied by elaborative waterpipe-specific didactic text with facts.

### Strengths and limitations

Our study is the first of its kind, thus our results may provide background evidence for further observational and experimental studies. We studied many measures of effectiveness that were not addressed collectively elsewhere. The rich literature available on effectiveness of PHWs concentrates on cigarette packs, while those on other tobacco products remain understudied [13]. Warning labels need to be tailored according to local cultural and product use contexts, though we assumed the main measures of warning label effectiveness are universal and comparable [35]. Therefore, we compared our results with previous cigarette warning research and found many similarities in effectiveness results. Yet, we suggested more waterpipe-specific indicators of smoking behaviour change, such as the reduction in hagar consumption. There were some limitations related to the study design and sampling method. The cross-sectional nature of the study design limits the generalizability of results and does not allow us to attribute causality for the observed associations. The purposive sampling method may limit the study’s external validity, however, we used nationally representative proportions for age, gender, residence and WTS status. The existing PHWs on WTPs studied were not waterpipe-specific; in fact, waterpipe-specific warnings have not been developed to date in tobacco control. Therefore, the existing generic PHWs on WTPs may not be impacting waterpipe tobacco smokers as intended. For example, comparative risk perception of waterpipe tobacco versus cigarettes was not addressed by the existing warnings, which was reflected in the minimal awareness of waterpipe smokers in our study about the nicotine content of WTPs. Also, other sources of knowledge about health risks of WTS or nicotine contents of waterpipe tobacco may have affected risk perceptions of participants and is important to consider when thinking about the motivational nature of the warning labels in behavior outcomes. The measures of warning effectiveness have not been used previously in a waterpipe-specific context, therefore, further validation of the measures used in our study is warranted. Also, social desirability may be biasing the results because we relied on self-reported changes in WTS-relevant knowledge and behaviour and the face-to-face interview approach used in administering the survey questionnaire. Adult female WTS has been reported to be less than 1% [11,12]; thus, it was expected to find a small number of female waterpipe smokers. Some tobacco control experts consider that there has been under-reporting of female WTS. We think it likely that they did not want their WTS status to be known, therefore, female waterpipe smokers were difficult to reach in our study and they were interviewed mainly at waterpipe serving cafes. Nonetheless, the large sample, the variety of responses from different population sources and response rates over 90%, allowing for sufficient observations among the compared subgroups, may have minimized potential biases in our results.

## Conclusions

This is the first study to measure recall of existing PHWs on WTPs and their effectiveness in encouraging quit-related behaviour in waterpipe smokers and preventing uptake in non-smokers, comparing these outcomes across socio-demographic groups. About two-thirds of waterpipe smokers and non-smokers noticed PHWs on WTPs. More than three-quarters of participants reported communication of health risks of WTS through the warnings, except for nicotine contents. At least half of participants cognitively processed PHWs on WTPs; non-smokers reported significantly higher rates of cognitive processing of WTP PHWs than waterpipe smokers, except for avoidance of warnings.

Participants who were aware of PHWs on WTPs found them helpful according to their self-reported behavioural responses: About two-thirds of waterpipe smokers reported that PHWs on WTPs helped them to quit and encouraged a similar proportion to reduce their consumption; about two-fifths reported they forgone a smoke due to viewing PHWs on WTPs; and a quarter reported that PHWs on WTPs helped them try to quit at least once. More than half of former waterpipe smokers reported that PHWs on WTPs helped them to quit and maintain long-term abstinence. About two-thirds of non-smokers reported that PHWs on WTPs helped in deterring them from WTS uptake.

Our findings suggest that inserting PHWs on WTPs is an effective waterpipe tobacco labelling policy. The results of our study are exploratory and provide background evidence of differences in effectiveness of WTP PHWs among socio-demographic groups. Future longitudinal observational and experimental research is needed to build evidence and inform a more effective waterpipe tobacco labelling policy in this sparsely studied area. Countries with similarly high rates of WTS should consider adopting PHWs on WTPs within a comprehensive regulatory framework.
